# Invasive Lobular Carcinoma Metastasis to the Female Genital Tract: A Systematic Search and Review of Case Reports and Case Series

**DOI:** 10.3390/diagnostics15182356

**Published:** 2025-09-17

**Authors:** Nektarios I. Koufopoulos, Abraham Pouliakis, Menelaos G. Samaras, Kostantinos Skarentzos, Theofanis Nastos, Ioannis Boutas, Adamantia Kontogeorgi, Magda Zanelli, Andrea Palicelli, Maurizio Zizzo, Giuseppe Broggi, Rosario Caltabiano, Serena Salzano, Dimitrios Goutas, Ioannis S. Pateras, John Syrios, Amanda Psyrri, Nikolaos Arkadopoulos, Ioannis G. Panayiotides

**Affiliations:** 1 Second Department of Pathology, Medical School, National and Kapodistrian University of Athens, Attikon University Hospital, 12462 Athens, Greece; apouliak@med.uoa.gr (A.P.); menelaos.g.samaras@gmail.com (M.G.S.); k.skarentzos@gmail.com (K.S.); fanis_nastos@hotmail.com (T.N.); dimgoutas@med.uoa.gr (D.G.); ipateras@med.uoa.gr (I.S.P.); ioagpan@med.uoa.gr (I.G.P.); 2 Breast Unit, Rea Maternity Hospital, P. Faliro, 17564 Athens, Greece; drboutas@gmail.com; 3 Third Department of Obstetrics and Gynecology, Medical School, National and Kapodistrian University of Athens, Attikon University Hospital, 12462 Athens, Greece; ad.kontogewrgi@gmail.com; 4 Pathology Unit, Azienda USL-IRCCS di Reggio Emilia, 42123 Reggio Emilia, Italy; andrea.palicelli@ausl.re.it; 5 Clinical and Experimental Medicine PhD Program, University of Modena and Reggio Emilia, 41121 Modena, Italy; maurizio.zizzo@ausl.re.it; 6 Surgical Oncology Unit, Azienda USL-IRCCS di Reggio Emilia, 42122 Reggio Emilia, Italy; 7 Department of Medical and Surgical Sciences and Advanced Technologies “G.F. Ingrassia” Anatomic Pathology, University of Catania, 95123 Catania, Italy; giuseppe.broggi@phd.unict.it (G.B.); rosario.caltabiano@unict.it (R.C.); sere.salzano@gmail.com (S.S.); 8 Second Department of Medical Oncology, Mitera Hospital, 15123 Athens, Greece; isyrios@hygeia-group.com; 9 Medical Oncology Unit, 2nd Department of Internal Medicine-Propaedeutic, Medical School, National and Kapodistrian University of Athens, Attikon University Hospital, Chaidari, 12462 Athens, Greece; psyrri237@yahoo.com; 10 4th Department of Surgery, Medical School, National and Kapodistrian University of Athens, Attikon University Hospital, Chaidari, 12462 Athens, Greece; narkado@hotmail.com

**Keywords:** breast carcinoma, invasive lobular carcinoma, female genital tract, metastasis, diagnosis

## Abstract

Invasive lobular carcinoma (ILC) is the most common special type of breast carcinoma, accounting for 5–15% of all breast carcinoma cases. Its metastatic pattern differs from that of invasive breast carcinoma of no special type, with ILC metastases to the peritoneum, gastrointestinal tract, and female genital tract being more frequent. This literature review focuses on ILC cases with metastasis to the female genital tract (FGT). Searches were conducted in medical databases including PubMed, Scopus, and Web of Science, using specific keywords. Inclusion criteria centered on studies presenting one or more cases of patients with ILC metastasis to the FGT and English language publications. Exclusion criteria included articles that did not present original research findings, studies with insufficient data, and publications in languages other than English. A thorough analysis of 154 results from PubMed, 56 from Scopus, and 173 from Web of Science after the application of inclusion and exclusion criteria resulted in the inclusion of 54 manuscripts describing 61 cases. The demographic, clinicopathological, and therapeutic aspects of ILC metastases to the FGT were reviewed and the differential diagnosis and prognosis for each anatomic location in the FGT were discussed separately. Our analysis of the data showed that the restricted mean survival time was 186 ± 30.7 months and that a negative ER on a secondary tumor was found to be linked to worse patient survival rates. Also of note is the fact that in 37.7% of cases there was involvement of multiple FGT anatomic locations and in 36% of cases there were metastases in organs or anatomic locations other than the FGT. To our knowledge, our study is the only one to describe the features of patients with secondary FGT involvement from ILC.

## 1. Introduction

Invasive lobular carcinoma (ILC) is the commonest special subtype of invasive breast carcinoma, accounting for 5–15% of breast carcinomas [1], thus being second in frequency only to invasive breast carcinoma of no special type (IBCNST) according to the latest WHO classification of breast tumors [2]. It was first described by Foote and Stewart in 1941 [3]. Its morphological, immunohistochemical, clinical, radiological, and molecular characteristics differ from those of IBCNST. It may remain undetectable or present as a palpable tumor [4]. In imaging studies, it may be detected with difficulty and tumor size may occasionally be underestimated, resulting in positive surgical margins [5]. The metastatic pattern also differs from that of IBCNST, with ILC metastases to the peritoneum, gastrointestinal tract, and female genital tract (FGT) being more frequent [6,7,8]. ILC lacks E-cadherin immunopositivity and displays aberrant β-catenin immunostaining [9]. Its molecular profile is characterized by deleterious mutations in CDH1 paired with allelic loss of the remaining allele [5].

Histologically, ILC is characterized by small cells with discohesive growth patterns forming single-cell files, and a minimal stromal response. Signet ring-like cells may sometimes be found [5]. Apart from the classic variant, several histological variants have been described, including alveolar, tubulolobular, solid, trabecular, signet ring, pleomorphic, and mixed [10,11,12,13,14,15]. Another three rare variants (ILC with extracellular mucin production, ILC with papillary features, and ILC with tubular elements) have been described recently [4].

We herewith review case reports and case series describing ILC metastases to the FGT. We also analyze the demographic, clinicopathological, and therapeutic aspects of ILC metastases to the female genital tract, and we discuss separately the differential diagnosis and prognosis for each anatomic location in the FGT.

## 2. Materials and Methods

### 2.1. Search Strategy

A literature review was conducted using PubMed, Scopus, and Web of Science to identify all published cases in the English language of ILC metastasis to the FGT. The research utilized the following terms: “lobular carcinoma” AND “metastasis” AND “female genital tract” OR “ovary” OR “ovarian” OR “vulva” OR “vagina” OR “endometrium” OR “endometrial” OR “uterus” OR “uterine”. We did not set any additional limitations while performing the search.

### 2.2. Inclusion and Exclusion Criteria

Two authors [MGS, KS] performed the literature review and collected data. Discrepancies were corrected by consensus. In cases where consensus could not be reached, the principal investigator (NK) resolved the disagreement.

The timeline for the selected studies ranged from October 1993 to April 2024.

Both reports with a single case and studies reporting at least two cases of ILC metastases to the FGT were included in the review. At the same time, we excluded narrative or systematic reviews, meta-analyses, opinion pieces, and other articles that did not present original research findings.

Papers available only as abstracts or those with text that was too brief or non-informative were excluded from the present review.

The clinicopathological and treatment parameters analyzed included age (median and range), clinical presentation, primary tumor size, ER, PR, and HER-2 status, both in primary focus and in the metastasis, tumor stage, location of metastatic involvement, metastases in organs or locations other than the FGT, surgical and neoadjuvant or adjuvant treatment, time interval to metastasis, median follow-up, outcome, and tumor grade.

In addition, cases with insufficient or too much aggregated data, as well as manuscripts in languages other than English, were excluded.

After applying inclusion and exclusion criteria, 54 manuscripts describing 61 cases of ILC with metastasis to the female genital tract [16,17,18,19,20,21,22,23,24,25,26,27,28,29,30,31,32,33,34,35,36,37,38,39,40,41,42,43,44,45,46,47,48,49,50,51,52,53,54,55,56,57,58,59,60,61,62,63,64,65,66,67,68,69] remained for data extraction.

### 2.3. Statistical Analysis

As detailed patient characteristics were available in the studied case reports, it was feasible to perform statistical analysis. Specifically, the descriptive characteristics of the quantitative data were expressed as median, Quartile 1 (Q1) to Quartile 3 (Q3), range, and, for completeness reasons mean ± standard deviation (SD). For the qualitative data, the frequency of occurrence and the relevant percentage were reported. It was also possible to evaluate overall survival (OS) via the Kaplan–Meier estimator and perform comparisons of OS with various characteristics via the log-rank method. The statistical analysis was performed using the R language for statistical analysis (version 4.4.0), and the significance level (*p*-value) was set to 0.05 when applicable tests were two-sided.

## 3. Results

### 3.1. Patient Characteristics

In total, 61 patients were reported in the studied case reports and case series. The mean patient age was 57.4 ± 12.2 years (min: 32; max: 86). The detailed descriptive statistics for patient characteristics are presented in Table 1.

### 3.2. Demographic and Clinicopathological Features

Metastasis of ILC to the FGT is uncommon. We were able to retrieve 54 manuscripts describing a total of 61 cases of ILC FGT secondaries. Primary tumor size was mentioned in 22/61 (36%) [17,18,21,22,23,25,28,32,34,36,39,40,41,43,50,53,58,59,63,65,66] cases. The mean tumor size was 36.5 mm (range 9–100 mm). The metastatic site was mentioned in all cases. Some of the patients had more than one FGT metastatic site. The most common metastatic site was the uterine corpus in 30/61 (49.2%) [20,24,27,28,30,35,36,38,39,40,41,43,44,45,46,47,49,50,51,53,54,55,56,59,61,62,64,65,66] patients, followed by the uterine cervix in 25/61 (41%) [21,22,24,27,28,30,31,32,34,35,39,44,46,50,53,54,58,59,62,65,66], and the ovary in 22/61 (36%) [28,29,30,35,36,38,42,44,48,51,53,54,57,63,65,66,67] patients. In 9/61 (14.7%) [16,20,26,27,52,60,68] cases, the metastatic site was an endometrial polyp, in 8/61 (13.1%) [17,23,27,33,37,38,44,45,52,59] a uterine leiomyoma, and in the vulva in 4/61 (6.5%) [18,25,37,42]. Less common metastatic sites were an ovarian granulosa cell tumor [19], the vagina [54], and an ovarian fibroma [69]. Metastases to sites other than the FGT were documented in 21/61 patients (34.4%) [18,20,21,23,36,37,38,40,41,42,43,44,45,46,49,57,59,62,63,64,66,67]. Metastatic spread to the bones was found in 12/21 (57.1%) [18,20,23,38,41,43,49,59,62,63,64,66] patients. Other metastatic sites were the pancreas [21], stomach [21,37,47,67], liver [36,38], pleura [37], peritoneum [37,42,43], lymph nodes [42,43,46], gallbladder [43], omentum [44], orbit [49], large bowel [57], and appendix [57].

Symptoms were mentioned in 60/61 (98.4%) [16,17,18,19,20,21,22,23,24,25,26,27,28,29,30,31,32,33,34,35,36,37,38,39,40,41,42,43,44,45,46,47,48,49,50,51,52,53,54,55,56,57,58,59,60,61,62,63,64,65,66,67,68,69] cases. Bleeding from the genitalia was the commonest symptom, being reported in 24/60 (40%) [17,20,21,24,26,28,33,35,37,41,43,44,49,52,53,54,55,56,58,60,61,64,65,68] cases, followed by abdominal pain in 6/60 (10%) [19,22,57,59,60,69] patients, a mass in 5/60 (8.3%) [18,23,25,51] cases, abdominal distention in 3/60 (5%) [44,63,69] cases, and abdominal discomfort in 3/60 (%) [39,67] patients. Less common symptoms included abdominal fullness [29], loss of appetite [29], urinary incontinence [38], polyuria [39], abdominal bloating [42,57], abdominal compression [45], postcoital bleeding [46], vaginal fullness and discomfort [51], altered bowel habits [57], and right shoulder pain [66] in 1/60 (1.7%) of patients each. Finally, 14/60 (23.3%) [16,27,30,31,32,34,36,40,47,48,49,50,51,62] patients were asymptomatic. In 42 cases, the FGT metastasis was metachronous, while in 12 cases it was concurrent with the primary tumor. The interval to metastasis ranged from 2 to 360 months (mean 65.6 months). The detailed demographic and clinicopathological features of the cases are shown in Table 2.

### 3.3. Histological Findings

No information regarding the subtype of ILC was available, apart from one case of ILC with extracellular mucin production [69], which is a very rare subtype with around forty cases in the English literature [1,3,4].

### 3.4. Estrogen Receptors (ER)/Progesterone Receptors (PR)/HER-2 Status

Concerning hormonal and HER-2 status, 36/61 (59%) [18,20,21,23,29,30,31,32,33,34,35,36,37,38,39,40,41,42,43,44,45,46,47,48,50,54,55,56,58,59,63,65,66,68] cases reported ER and PR in the primary focus and 34/61 (55.7%) [18,19,20,21,23,26,27,28,29,32,35,37,39,40,41,42,44,45,46,47,48,50,52,56,57,58,59,62,63,64,66,68] in the metastatic setting. HER-2 status was reported in 24/61 (39.3%) cases [29,30,32,34,35,37,38,39,40,41,43,44,45,47,50,54,55,56,58,59,63,65,66,68] in the primary focus and in 12/61 cases (19.7%) [28,29,32,35,46,47,52,58,62,64] in the metastatic location. ER, PR, and HER-2 status of the cases and the histological grades are shown in Table 3.

### 3.5. Treatment

Surgical treatment information was available in 51/61 (83.6%) [17,18,19,20,21,22,23,24,25,26,27,28,29,30,31,32,33,34,35,36,37,38,39,40,41,42,43,44,45,46,47,48,49,52,54,55,56,57,58,59,61,62,63,64,65,66,67,68] cases. In 32/51 (62.7%) [17,20,21,22,23,25,26,27,28,29,30,31,35,39,40,42,44,47,49,54,55,56,57,58,61,62,65,66,67,68] cases, surgical treatment consisted of a modified radical mastectomy. Breast-conserving surgery was performed in 9/51 (17.6%) [18,32,34,36,41,45,46,48,53] cases, hysterectomy and bilateral salpingo-oophorectomy in 5/51 [23,36,39,59,63] (9.8%) cases, and breast biopsies in 3/51 (5.9%) [33,43,44] cases. Omentectomy was performed in 2/51 (3.9%) [36,63] cases. Endometrial and cervical biopsies were performed in 2/51 (3.9%) [34,49] cases, cervical biopsy in 1/51 (1.9%) [34] cases, and partial vulvectomy in 1/51 (1.9%) [18] cases. Additionally, two manuscripts reported that surgical treatment was performed without any additional detail concerning the type of intervention [52,64]. In cases with metachronous FGT metastasis, second-line treatment was reported in 43/49 (87.7%) [16,19,20,21,22,23,25,27,28,29,30,31,32,33,35,37,38,40,41,42,44,45,46,47,48,49,52,53,54,55,56,57,58,60,61,62,64,65,66,68] cases. Surgical treatment involved hysterectomy and bilateral salpingo-oophorectomy in 22/43 (51.2%) [16,21,26,28,29,30,32,35,38,41,42,45,48,49,52,53,54,55,58,60,64,65,66,68] patients, bilateral oophorectomy in 2/43 (4.6%) [19,57] cases, pancreatoduodenectomy in 1/43 (2.3%) [21] cases, wide tumor excision in 1/43 (2.3%) [25] cases, omentectomy in 4/43 (9.3%) [29,30,48,64] cases, pelvic lymph node sampling in 1/43 (2.3%) [29] cases, pelvic lymphadenectomy in 2/43 (4.6%) [30,32] cases, excision of endometrial polyps in 2/43 (4.6%) [33,37] cases, appendectomy in 2/43 (4.6%) [42,57] cases, endometrial biopsy in 1/43 (2.3%) [47] cases, peritoneal biopsies in 2/43 (4.6%) [48,64] cases, partial colectomy in 1/43 (2.3%) [52] cases, and anterior resection in 1/43 (2.3%) [57] cases. Biopsy of the metastatic lesion was performed in 5/43 (11.6%) cases [20,46,56,61,62].

Information regarding adjuvant treatment was provided in 50/61 (83.6%) [17,19,20,21,22,23,25,26,27,28,29,30,31,32,33,34,35,36,37,38,39,40,41,42,43,44,45,46,47,48,49,50,52,53,54,55,56,57,58,59,62,63,64,65,66,67,68] cases. Chemotherapy either in the adjuvant or neoadjuvant setting was offered in 43/50 (86%) [17,19,20,22,23,25,27,28,29,31,33,34,35,36,38,40,41,42,43,44,45,46,47,48,49,52,53,54,55,56,57,58,59,62,63,64,65,66,67,68] cases and radiotherapy in 27/50 (54%) [19,20,22,27,28,29,31,32,34,35,42,44,45,46,47,48,49,52,53,54,55,57,58,62,65,67,68] cases. In two cases, patients refused chemotherapy [30,50], and in another two, radiotherapy [30,66]. The most common regimen used consisted of cyclophosphamide, methotrexate, and 5-fluorouracil (CMF regimen). In contrast, the second most common regimen consisted of adriamycin, cyclophosphamide (AC regimen), and paclitaxel administered in four [20,25,31,42] and three cases [54,58,63], respectively. Hormonal treatment was provided to 48/50 (96%) [17,19,20,21,22,23,25,26,27,28,29,30,31,32,34,35,36,37,38,39,40,41,42,43,44,45,46,47,49,52,53,54,55,56,57,58,59,62,63,64,65,66,67,68] patients. Among 43 cases with metachronous metastasis, 13 received additional chemotherapy [21,25,30,32,40,42,44,46,47,48,54,56,66,67], one received targeted therapy [65], two received additional radiotherapy [23,41], and 13 received additional hormonal treatment [19,21,23,32,35,41,42,45,52,58,64,65,68].

### 3.6. Outcome

Follow-up information was available in 40/61 (65.6%) [17,18,19,21,22,23,25,28,30,31,32,34,35,36,37,38,39,40,41,42,43,45,47,48,50,51,52,53,54,58,60,62,63,64,66,67,68] cases. Briefly, 16/40 (40%) [17,19,32,35,36,37,38,39,45,48,52,53,58,60,64,67,68] patients were alive without evidence of disease, 9/40 (22.5%) [18,23,25,30,34,41,42,47,51] were alive with disease, and 10/40 (25%) [21,28,31,40,43,51,54,66] died of disease in a period of time that ranged from 1 to 308 months. In 2/40 (5%) [22,50] cases, patients were lost to follow-up. Treatment and follow-up data are shown in Table 4.

### 3.7. Patient Survival

**Figure 1:**
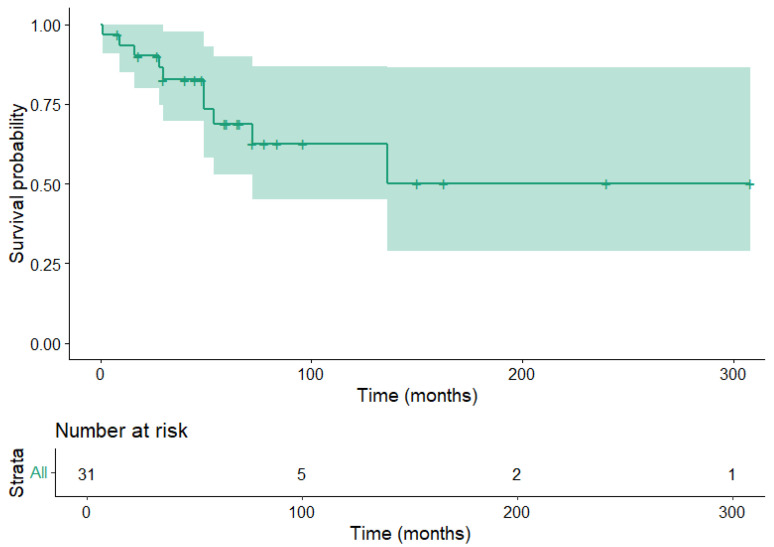
Kaplan–Meier estimator for patient overall survival. The shaded area corresponds to the 95% confidence interval.

Patient survival time information was available for 31 patients. Of these patients, 10 were deceased due to their disease. The restricted mean survival time was 186 ± 30.7 months. Kaplan–Meier curves for overall survival are shown in Figure 1.

**Figure 2:**
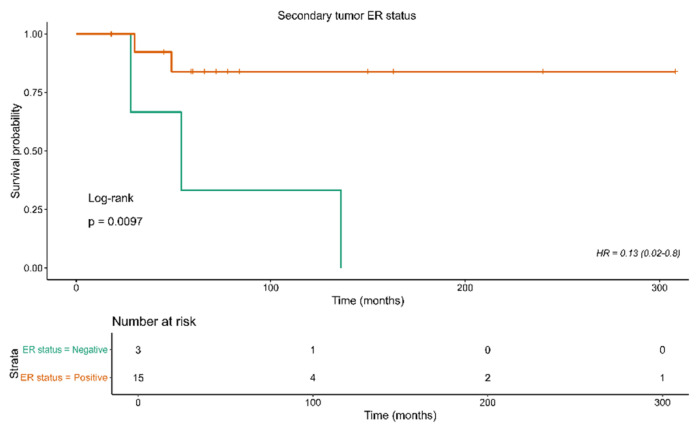
Kaplan–Meier curves for overall survival in relation to ER status of the secondary tumor.

Further analysis was based on the evaluation of the role of all recorded characteristics in patient survival. The results are depicted in Table 5. Notably, a negative ER on a secondary tumor was found to be linked to worse patient survival (HR: 0.13, 95% CI: 0.02–0.8, *p* = 0.01); see Figure 2.

## 4. Discussion

Extragenital metastases to the FGT are relatively uncommon. Concerning specific locations of secondaries, one study reported that, among 149 metastatic neoplasms to the FGT from primary extragenital tumors reported in one study, the ovary (75.8%) and vagina (13.4%) were the most frequent locations, followed by the endometrium (4.7%) and cervix (3.4%) [70]. In our review the most common metastatic location was the uterine corpus in 30 (49.2%) cases, followed by the uterine cervix in 25 (41%) patients, and the ovary in 22 (36%) patients. We believe this difference can be explained by the fact that we analyzed mostly case reports. We excluded from our search some cases series reporting ovarian metastases due to the fact that they did not mention the subtype of metastatic breast carcinoma. The majority of FGT metastases from breast cancer occur in advanced cases during hormonal treatment or follow-up [53]. Similarly, in several of our cases metastasis of ILC occurred while patients were under hormonal treatment.

In the literature, the incidence of ILC metastasis to the FGT ranges from 2% to 5% in clinical series [5] and from 36% to 52% in autopsy series [71,72]. This difference is probably due to the fact that autopsy may discover clinically occult micrometastases. In our review, there was involvement of more than one FGT site in 37.7% of cases, and in 36% of cases there were metastases in organs or anatomic locations other than the FGT. The most common locations were skeletal metastases, which occurred in more than half of these cases, followed by the stomach.

The ovaries frequently receive metastases from primary malignant tumors both of genital and extragenital sites [73,74]. This can be explained by the fact that they provide an excellent environment for malignant cell implantation due to their rich vasculature and extensive lymphatic network, as well as due to a favorable pH and oxygen pressure in the ovarian stroma [31]. In a number of studies, the most common primary site varies between the gastrointestinal tract and the breast [75]. Ovarian metastasis from breast carcinoma constitutes 3–38% of all ovarian neoplasms, with a variable incidence depending on diagnostic methods, geographic distributions, and other variables [76]. Studies have shown that breast cancer patients have an incidence of ovarian metastases of 13–47%, either in autopsy or surgical material [77,78]. ILC ovarian metastases usually manifest as bilateral solid and cystic masses, the so-called Krükenberg tumor(s). Micrometastatic disease may remain undetected both on clinical examination and in imaging studies [79].

Metastases in the ovaries may occasionally mimic the clinical and histological characteristics of primary ovarian carcinomas [73]. The distinction between metastatic versus primary ovarian carcinoma is of paramount importance since their management differs [72,75]. To date, there are no clear guidelines concerning the management of carcinomas metastatic to the ovary. However, surgical resection may increase patient survival rates [79].

Some clinicopathologic factors of primary breast carcinoma have been identified to be related to increased risk of ovarian metastasis. ILC has an increased metastatic potential to the ovaries [5]. Also, young age and premenopausal status are factors related to increased risk [80]. Other factors related to the development of ovarian metastases are other co-existent metastatic sites [81], large primary tumor size [82,83], inflammatory breast cancer [84], positive lymph nodes [85,86], higher stage (III-IV) [87], and bilaterality [81].

The incidental finding of an ovarian mass in an asymptomatic patient may be the first sign of ovarian metastasis [77]. Usually, they are bilateral, small, and solid [88,89]. Other symptoms, including gastrointestinal symptoms, ascites, pelvic pain, and vaginal bleeding, can be observed in some patients [77]. However, none of these clinical manifestations is related to either breast metastasis or primary ovarian carcinoma [90]. In our review, only 3/20 (15%) [30,48,51] cases were asymptomatic.

Imaging examinations are also widely used for the diagnosis, staging, and monitoring of curative effects.

The pathologic examination of a specimen includes gross, microscopic, and immunohistochemical testing. These are considered the ‘gold standard’ in the diagnosis of metastatic breast cancer to the ovaries [91].

On gross examination, bilateral involvement, small size, and a solid mass are clues related to metastatic breast carcinoma [92,93,94]. Metastases in the ovaries are usually located in the ovarian medulla and/or cortex [88]. On the other hand, primary tumors are typically located in the ovarian surface epithelium and superficial cortex [95].

Microscopically, ovarian metastases sometimes mimic histological features of primary ovarian carcinomas [96,97], which makes their distinction difficult. The characteristic pattern of ILC of small, discohesive cells forming single-cell files will usually allow diagnosis on hematoxylin and eosin stains. In difficult cases, immunohistochemical analysis typically resolves any diagnostic problem. Immunostaining for TRPS1, GATA-3, GCDFP-15, and mammaglobin favors metastasis of breast origin [98], whereas PAX-8, WT-1 p53, and p16 staining favors primary ovarian carcinoma. It is important to remember that mammaglobin can be expressed in gynaecologic malignancies [99].

Concerning treatment options, most breast cancer patients have other non-FGT metastases at the time of ovarian metastasis. The treatment should be for systemic disease. The regimen should be tailored to the clinicopathological aspects of the metastatic site, the burden of disease, the eventual visceral crisis, the symptoms, and the performance status of the patient. Drug toxicity profile and patient preferences are of utmost importance [85].

The prognosis of patients with breast carcinoma ovarian metastases is poor since the median progression-free survival ranges from 9 to 30 months, the median overall survival is 16 to 38 months, and the 5-year survival rate is 6 to 26% [79].

Clinicopathologic factors that affect survival are age [96], time interval to ovarian metastasis [82], unilaterality [100], and menstrual status [89].

Uterine metastases from extragenital cancers are much less common than ovarian metastases [52]. Metastases confined to the uterus, without ovarian involvement, are very rare and can occur through hematogenous spread [52]. The myometrium is the most commonly involved location within the corpus uteri that metastatic ILC involves, followed by the endometrium. The first manifestation of metastatic disease may be abnormal uterine bleeding [86]. ILC is, in most cases, ER-positive. Premenopausal patients regularly receive tamoxifen as part of the adjuvant treatment, which increases the risk for endometrioid carcinoma of the endometrium [86]. Differentiating metastatic ILC from a primary endometrial carcinoma can be difficult but is of huge importance since the treatment is different for these carcinomas.

Metastasis from breast carcinoma to the cervix uteri is very rare, with an estimated frequency of 0.8–1.7% [101]. This is possibly due to its small size, its reduced blood flow and distal circulation, and the presence of abundant fibrous tissue [102]. The true incidence of cervical metastasis from ILC remains unknown. Other distant metastases were found at the time of diagnosis of cervical metastasis in 67–89% of cases [103]. In our review, we found 22 cases of ILC metastatic to the cervix. Other distant metastases were found in only 27% of cases. Differential diagnosis between a cervical primary and ILC cervical metastasis may occasionally prove difficult. Dedifferentiation of cervical adenocarcinoma and squamous cell carcinoma with acantholytic changes may result in the simulation of the morphology of ILC [104]. Again, the finding of the characteristic morphology of ILC, i.e., discohesive cells and the formation of single cell files within the cervical stroma, especially when the cervical epithelium is spared, should raise the suspicion of metastatic disease. Appropriate immunohistostaining with TRPS-1, GATA3, mammaglobin, and GCDFP-15 will provide the diagnostic solution in ambiguous cases.

Vaginal metastasis is second in frequency after ovarian involvement in the FGT. Treatment consists of surgical debulking chemotherapy and/or radiotherapy. For patients with vaginal metastasis from breast carcinoma a very important prognostic feature is the finding of metastases in other sites [105]. A lot of times, when vaginal metastases occur, there are already metastases in other organs. Whenever this happens, the prognosis is poor [106]. In our review we found a single case of vaginal metastasis. This case had metastases also to the uterus, bilateral ovaries, and cervix. The patient succumbed to disease 16 months after diagnosis.

Breast cancer metastasis to the vulva is very rare. In these cases, the differential diagnosis is conducted with primary breast carcinoma of the vulva [107]. The most important distinguishing feature is previous history of breast cancer. Additionally, the histological similarity between the primary breast and the metastatic lesion, as well as the absence of an in situ element, will guide the pathologist in diagnosing a metastatic lesion [107].

Our study is unique due to the fact that it analyzes the very rare occurrence of FGT metastases of ILC. However, there are some limitations in our study. Missing information in several papers was frequently encountered. Missing data included hormone receptor status, detailed information on treatment in some cases, and information concerning survival. We propose that in the future, cases of ILC with FGT metastases from many different centers should be gathered, with sufficient demographic, clinical, pathological, treatment, and outcome information to draw more definitive conclusions regarding the prognosis of ILC metastasis to the FGT.

## 5. Conclusions

In summary, we reviewed cases and case series of ILC metastasis to the FGT, describing clinical, pathological, therapeutic, and follow-up data. We also discussed the current literature focusing on the differential diagnosis, treatment, and prognosis of ILC metastasis to the FGT. Our review showed that the most common site of FGT metastasis from ILC was the uterus followed by the uterine cervix, a finding that is not in line with previous studies. Involvement of more than one FGT site was present in 37.7% of cases, and metastases in locations other than the FGT were present in 36% of cases. These findings show that FGT metastasis from ILC occurs frequently in the setting of disseminated disease.

## Figures and Tables

**Table 1:** Descriptive characteristics of the reported cases in the studied case reports. ANED—alive, no evidence of disease; AWD—alive with the disease; DOD—died of disease; SD—standard deviation.

Characteristic	Measure
**Number of cases**	61
**Age**	
Mean ± SD	57.4 ± 12.2
Median [Min, Max]	56.0 [32.0, 86.0]
**Tumor Size (cm)**	
Mean ± SD	3.65 ± 2.11
Median [Min, Max]	3.65 [0.900, 10.0]
Missing	39 (63.9%)
**Primary tumor ER status**	
Negative	2 (3.3%)
Positive	34 (55.7%)
Missing	25 (41.0%)
**Primary tumor PR status**	
Negative	2 (3.3%)
Positive	34 (55.7%)
Missing	25 (41.0%)
**HER-2 status**	
Negative	21 (34.4%)
Positive	3 (4.9%)
Missing	37 (60.7%)
**Secondary tumor ER status**	
Negative	3 (4.9%)
Positive	31 (50.8%)
Missing	27 (44.3%)
**Secondary tumor PR status**	
Negative	6 (9.8%)
Positive	24 (39.3%)
Missing	31 (50.8%)
**HER-2 status**	
Negative	9 (14.8%)
Positive	3 (4.9%)
Missing	49 (80.3%)
**Stage pT component**	
pT1b	1 (1.6%)
pT1c	6 (9.8%)
pT2	16 (26.2%)
pT3	6 (9.8%)
pT4	1 (1.6%)
Missing	31 (50.8%)
**Stage pN component**	
pN0	8 (13.1%)
pN1	10 (16.4%)
pN2	5 (8.2%)
pN3	9 (14.7%)
Missing	29 (47.5%)
**Stage M component**	
M0	22 (36.1%)
M1	11 (18%)
Missing	27 (54.1%)
**Radiotherapy**	
No	24 (39.3%)
Yes	27 (44.3%)
Missing	10 (16.4%)
**Interval to Metastasis (in months)**	
Mean ± SD	65.6 ± 70.0
Median [Min, Max]	48.0 [2.00, 360]
Missing	20 (32.8%)
**Last follow up (in months)**	
Mean ± SD	71.1 ± 67.6
Median [Min, Max]	54.0 [1.00, 308]
Missing	30 (49.2%)
**Last follow up status**	
ANED	19 (31.1%)
AWD	9 (14.8%)
DOD	10 (16.4%)
Missing	23 (37.7%)
**Tumor grade**	
Grade II	10 (16.4%)
Grade III	2 (3.3%)
Missing	49 (80.3%)

**Table 2:** Demographic and clinicopathological features of the reported cases.

Study	Year	Age	Clinical Presentation	Tumor Size (cm)	Stage	Site of Metastasis FGT	Other Metastatic Sites
Aranda et al. [16]	1993	76	Asymptomatic	NM	pTx, N0	Endometrial polyps	-
Sugiyama et al. [17]	1995	51	Hypermenorrhea	4	pT2, N1, M1	Uterine leiomyoma	-
Menzin et al. [18]	1998	53	Vulvar tumor	2.5	pT2, N0, M1	Vulva	Vertebrae and pelvic bones
Arnould et al. [19]	2002	59	Abdominal and pelvic pain	-	-	Ovarian granulosa cell tumor	-
Alvarez et al. [20]	2003	69	Metrorrhagia	-	pT2, N1, M0	Endometrial polyps and uterus	Skull and spine
Ogino et al. [21]	2003	49	Abnormal genital bleeding	3.5	pT2, N0, M0	Cervix	Pancreas, stomach
Rau et al. [22]	2003	55	Abdominal pain	5	pT4, N1, M0	Cervix	-
Blecharz et al. [23]	2004	46	Enlarged uterus	1	pT1c, N2	Uterine leiomyoma	Bone
Famoriyo et al. [24]	2004	78	Postmenstrual bleeding	-	NM	Uterus and cervix	-
Sheen-Chen et al. [25]	2004	32	Vulvar tumor	3	pT2, N0, M0	Vulva	-
Al-brachim et al. [26]	2005	53	Vaginal bleeding	NM	pTxN1	Endometrial polyps	-
Lee et al. [27]	2005	76	Asymptomatic	NM	NM	Uterine leiomyoma, myometrium, endometrial polyps, cervical stroma, and soft tissue adjacent to the uterus and the cervix	-
Scopa et al. [28]	2005	50	Vaginal bleeding	6	pT3N3	Endometrium, endocervix, ovaries, and fallopian tubes.	-
Scopa et al. [28]	2005	81	Vaginal bleeding	1.6	pT1c, N3	Endometrium	-
Chen et al. [29]	2006	47	Poor appetite and abdominal fullness		pT3, N0, M0	Ovary	-
Erkanli et al. [30]	2006	63	Asymptomatic	NM	pT2, N3, M0	Endometrium, cervix, and ovaries.	-
Perisic et al. [31]	2007	65	Asymptomatic	NM	pT2, N1, M0	Cervix	-
Manci et al. [32]	2008	41	Asymptomatic	1	pT1c, N0, M0	Cervix	-
Manipadan et al. [33]	2008	70	Vaginal bleeding	NM	NM	Endometrial polyps	-
Bogliolo et al. [34]	2009	78	Asymptomatic	0.9	pT1b, N2, M1	Cervix	-
Ustaalioglu et al. [35]	2009	56	Vaginal bleeding	NM	pT2, N2, M0	Endometrium, myometrium, cervix, left uterine tube and left ovary.	-
Engelstaedter et al. [36]	2011	64	Asymptomatic	4	pT3, N3, M1	uterus, adnexa	Liver
Hooker et al. [37]	2011	83	Postmenopausal uterine bleeding	NM	NM	Endometrial polyps and vulva	Pleura, peritoneum, stomach
Isci et al. [38]	2011	48	Increased abdominal girth urinary incontinence	NM	NM	Both the ovaries, tubes, abdominal washing fluid, myometrium, and the huge leiomyoma	Liver, bone
Horikawa et al. [39]	2012	52	abdominal discomfort, polyuria.	5	pT2, N3, M1	Cervix and endometrium	-
Komeda et al. [40]	2012	59	Asymptomatic	3.8	pT3, N2, M0	Uterus	Multiple metastases
Vicioso et al. [41]	2013	67	Metrorrhagia	3.2	pT2, N2, M0	Uterus	Left femur, calvarial skull, axial skeleton, and rib cage
Alligood-Percoco et al. [42]	2015	36	Abdominal bloating	NM	NM	Ovaries/vulva	Peritoneum, lymph nodes
Bezpalko et al. [43]	2015	47	Vaginal bleeding	1.8	pT1c, Nx, M1	Endometrium	Gallbladder, bone marrow, lymph nodes, and peritoneum
Lokadasan et al. [44]	2015	49	Menorrhagia	NM	NM	Endometrium, myometrium, fibroid, cervix, and bilateral ovaries.	-
Lokadasan et al. [44]	2015	49	Abdominal distention, pain	NM	pT3, N3, M0	Cervix and bilateral adnexa	Omentum
Toyoshima et al. [45]	2015	62	Abdominal compression.	NM	pT2, N1, M0	Uterine leiomyomata, and myometrium	-
Waks et al. [46]	2015	53	Postcoital bleeding	NM	pT2, N1, M0	Cervix and corpus uteri	Pelvic and para-aortic lymph nodes
Lai et al. [47]	2016	54	Asymptomatic	NM	pT3, N3, M0	Endometrium	Stomach
Makris et al. [48]	2016	45	Asymptomatic	NM	pT2, N1, M0	Ovary	-
Martinez et al. [49]	2016	40	Vaginal bleeding	NM	NM	Endometrium	Orbit and bone
Martinez et al. [49]	2016	48	Asymptomatic	NM	NM	Endometrium and myometrium	-
Akhtar et al. [50]	2017	62	Asymptomatic	2.9	NM	Endometrium and cervix	-
Bennett et al. [51]	2017	53	Asymptomatic	NM		Bilateral ovaries	-
Bennett et al. [51]	2017	64	Vaginal fullness and discomfort	NM		Bilateral ovaries, uterus, and fallopian tubes	-
Bennett et al. [51]	2017	54	Adnexal mass	NM		-	-
Bennett et al. [51]	2017	41	Adnexal mass	NM		Bilateral ovaries and fallopian tube	-
Bennett et al. [51]	2017	48	NM	NM		Left ovary and uterine serosa	-
Razia et al. [52]	2017	58	Abnormal uterine bleeding	NM	NM	Endometrial polyps, uterine leiomyoma, and cervix	-
Seo et al. [53]	2017	46	Vaginal bleeding	4	pT2, N0, M0	Uterine corpus, endocervix, and left ovary	-
Aytekin et al. [54]	2018	38	Vaginal bleeding	NM	pT2, N3, M0	Uterus, bilateral ovaries, vaginal cuff, and cervix	-
Briki et al. [55]	2018	50	Postmenopausal uterine bleeding	NM	pT2, N1, M0	Endometrium	-
Franko-Marquez et al. [56]	2019	86	Abnormal uterine bleeding	NM	NM	Endometrium	-
Kachi et al. [57]	2019	58	Altered bowel habits, abdominal pain, and bloating	NM	NM	Bilateral ovaries	large bowel, appendix
Fontinele et al. [58]	2019	57	Abnormal uterine bleeding	10	ypT0, N0, M0	Cervix	-
Abdallah et al. [59]	2020	59	Lower abdominal pain	5	NM	Endometrium, myometrium, leiomyoma, and cervix	Scapula
Arif et al. [60]	2020	55	Vaginal bleeding, lower abdominal pain.	NM	NM	Endometrial polyp	-
Gomez et al. [61]	2020	69	Post-menopausal bleeding	NM	NM	Endometrium	-
Yuan et al. [62]	2020	64	Asymptomatic	NM	NM	Endometrium and cervix	Bones
Akizawa et al. [63]	2021	49	Abdominal distention	5	NM	Ovary	Bones
Awazu et al. [64]	2021	66	Abnormal genital bleeding	NM	NM	Endometrium	Bones
Lim et al. [65]	2021	57	Vaginal bleeding	5.6	NM	Uterus, cervix, bilateral ovaries, and fallopian tubes	-
Kong et al. [66]	2022	64	Right shoulder pain	1.5	pT1c, N3 M0	Uterus, cervix, bilateral ovaries, and fallopian tubes	Bones
Li et al. [67]	2022	61	Stomach discomfort	NM	pT1c, N1, M0	Bilateral ovaries and peritoneum	Stomach
Benlghazi et al. [68]	2024	56	Abnormal uterine bleeding	NM	NM	Endometrial polyps	-
Faur et al. [69]	2024	82	Abdominal distension and pain	NM	NM	Ovarian fibroma	-

Abbreviations: FGT—female genital tract; HBSO—hysterectomy and bilateral salpingo-oophorectomy; Met—metastasis; Mo—months; N—No; Neoadj.—neoadjuvant; NM—not mentioned; OM—omentectomy; RT—radiotherapy; Y—Yes.

**Table 3:** ER, PR, and HER-2 status and histological grades of the reported cases.

Study	Primary Tumor			Metastatic Tumor			Tumor Grade
	ER Status	PR Status	HER-2 Status	ER Status	PR Status	HER-2 Status	
Aranda et al. [16]	NM	NM	NM	NM	NM	NM	NM
Sugiyama et al. [17]	NM	NM	NM	NM	NM	NM	NM
Menzin et al. [18]	+	+	NM	+	+	NM	NM
Arnould et al. [19]	NM	NM	NM	+	+	NM	NM
Alvarez et al. [20]	+	+	NM	+	+	NM	NM
Ogino et al. [21]	−	+	NM	−	+	NM	NM
Rau et al. [22]	NM	NM	NM	NM	NM	NM	NM
Blecharz et al. [23]	+	+	NM	+	−	NM	NM
Famoriyo et al. [24]	NM	NM	NM	NM	NM	NM	NM
Sheen-Chen et al. [25]	NM	NM	NM	NM	NM	NM	NM
Al-brachim et al. [26]	NM	NM	NM	+	+	NM	NM
Lee et al. [27]	NM	NM	NM	+	+	NM	Grade II
Scopa et al. [28]	−	+	NM	−	+	+	NM
Scopa et al. [28]	NM	NM	NM	+	+	NM	NM
Chen et al. [29]	+	+	−	+	+	−	Grade III
Erkanli et al. [30]	+	+	−	NM	NM	NM	−
Perisic et al. [31]	+	+	NM	NM	NM	NM	Grade II
Manci et al. [32]	+	+	−	+	+	+	NM
Manipadan et al. [33]	Not done	Not done	Not done	NM	NM	NM	NM
Bogliolo et al. [34]	+	+	−	NM	NM	NM	Grade II
Ustaalioglu et al. [35]	+	+	−	+	+	−	NM
Engelstaedter et al. [36]	+	+					Grade II
Hooker et al. [37]	+	+	−	+	−	NM	NM
Isci et al. [38]	+	−	−	NM	NM	NM	NM
Horikawa et al. [39]	+	+	−	+	NM	NM	NM
Komeda et al. [40]	+	+	−	−	−	NM	NM
Vicioso et al. [41]	+	+	−	+	−		Grade II
Alligood-Percoco et al. [42]	+	+	NM	+	NM	NM	NM
Bezpalko et al. [43]	+	+	−	NM	NM	NM	Grade II
Lokadasan et al. [44]	+	+	−	NM	NM	NM	NM
Lokadasan et al. [44]	+	+	NM	+	+	NM	Grade II
Toyoshima et al. [45]	+	+	+	+	−	NM	NM
Waks et al. [46]	+	+		+	+	−	NM
Lai et al. [47]	+	+	−	+	+	−	Grade II
Makris et al. [48]	+	+	NM	+	+	NM	NM
Martinez et al. [49]	NM	NM	NM	NM	NM	NM	
Martinez et al. [49]	NM	NM	NM	NM	NM	NM	NM
Akhtar et al. [50]	+	+	−	+	+	NM	NM
Bennett et al. [51]	NM	NM	NM	NM	NM	NM	NM
Bennett et al. [51]	NM	NM	NM	NM	NM	NM	NM
Bennett et al. [51]	NM	NM	NM	NM	NM	NM	NM
Bennett et al. [51]	NM	NM	NM	NM	NM	NM	NM
Bennett et al. [51]	NM	NM	NM	NM	NM	NM	NM
Razia et al. [52]	NM	NM	NM	+	+	+	NM
Seo et al. [53]	+	+	NM	+	+	NM	NM
Aytekin et al. [54]	+	+	−	NM	NM	NM	NM
Briki et al. [55]	+	+	−	NM	NM	NM	NM
Franko-Marquez et al. [56]	+	+	+	+	NM	NM	NM
Kachi et al. [57]	NM	NM	NM	+	+	NM	NM
Silva Fontinele et al. [58]	+	+	−	+	+	−	Grade II
Abdallah et al. [59]	+	+	−	+	+	NM	NM
Arif et al. [60]	NM	NM	NM	NM	NM	NM	NM
Gomez et al. [61]	NM	NM	NM	NM	NM	NM	Grade II
Yuan et al. [62]	NM	NM	NM	+	+	−	NM
Akizawa et al. [63]	+	+	−	+	NM	NM	NM
Awazu et al. [64]	NM	NM	NM	+	+	−	NM
Lim et al. [65]	+	+	+	NM	NM	NM	NM
Kong et al. [66]	+	−	−	+	+	−	NM
Li et al. [67]	NM	NM	NM	NM	NM	NM	NM
Benlghazi et al. [68]	+	+	−	+	−	−	Grade III
Faur et al. [69]	NM	NM	NM	NM	NM	NM	NM

Abbreviations: ER—estrogen receptors; NM—not mentioned; PR—progesterone receptors.

**Table 4:** Treatment and follow-up features of the reported cases.

Study	Surgery	CHT	RT	Hormonal Therapy	Interval to Met (Mo)	Second-Line Therapy	Outcome
Aranda et al. [16]	NM	NM	NM	NM	36	HBSO	NM
Sugiyama et al. [17]	MRM	5-FU, mitomycin C, and pirarubicin	N	Tamoxifen	Concomitant	-	48 ANED
Menzin et al. [18]	Quadrantectomy, ALND, and partial vulvectomy.	NM	N	Tamoxifen	Concomitant	-	18 AWD
Arnould et al. [19]	NM	CHT	Y	Tamoxifen	48	Bilateral oophorectomy and letrozole	60 ANED
Alvarez et al. [20]	MRM	6 × CMF	Y	Tamoxifen	48	Biopsy	NM
Ogino et al. [21]	MRM	No	N	Tamoxifen	128	Pancreatoduodenectomy, HBSO, ADM-TXL CHT, and anastrozole.	136 DOD
Rau et al. [22]	MRM	6 × CHT	Y	Tamoxifen	48	Patient refused	Lost to follow-up
Blecharz et al. [23]	MRM, HBSO	6 × ADR, CTX, 5-FU.	N	Tamoxifen	Concomitant	Aromatase inhibitors, bisphosphonates, and palliative radiotherapy of the thoracic and lumbar vertebrae	59 AWD
Famoriyo et al. [24]	NM	NM	NM	Tamoxifen	NM	NM	NM
Sheen-Chen et al. [25]	MRM	6 × CMF	N	Tamoxifen	40	Wide excision of the tumor, cyclophosphamide, epirubicin, and 5-FU.	40 AWD
Al-brachim et al. [26]	MRM	No	N	Tamoxifen	48	NM	NM
Lee et al. [27]	MRM	CHT	Y	Tamoxifen	18	HBSO	
Scopa et al. [28]	MRM	4 × epirubicin	Y	Tamoxifen and LH-RH agonist	36	HBSO	54 DOD
Scopa et al. [28]	MRM	4 × cyclophosphamide and adriamycin.	N	Tamoxifen and LH-RH agonist	24	HBSO	30 DOD
Chen et al. [29]	MRM	6 × CHT	Y	Tamoxifen	56	HBSO, partial OM, and pelvic lymph node sampling	NM
Erkanli et al. [30]	MRM	Patient refused CHT	Patient refused RT	-	8	HBSO, omentectomy, and pelvic lymphadenectomy. Cyclophosphamide, epirubicin, and 5-FU.	8 AWD
Perisic et al. [31]	MRM	6 × CMF	Y	Tamoxifen	52	The patient refused CHT	72 DOD
Manci et al. [32]	Quadrantectomy	No	Y	Tamoxifen	130	HBSO, pelvic lymphadenectomy, CHT, and Femara	150 ANED
Manipadan et al. [33]	Biopsy	6 × docetaxel and zoledronic acid	N	No	2	Polypectomy	NM
Bogliolo et al. [34]	Quadrantectomy, SLNB, endometrial and cervical biopsy.	6 × 5-FU, epirubicin, cyclophosphamide, and docetaxel	Y	Letrozole	Concomitant	-	30 AWD
Ustaalioglu et al. [35]	MRM	4 × doxorubicin, cyclophosphamide/4 × docetaxel	Y	Anastrozole	36	HBSO, exemestane	45 ANED
Engelstaedter et al. [36]	HBSO, OM, lumpectomy, ALND.	Navelbine	N	Tamoxifen	Concomitant	-	65 ANED
Hooker et al. [37]	No	No	N	Letrozole, tamoxifen, fulvestrant	60	Polypectomy	72 ANED
Isci et al. [38]	No	CHT	N	Letrozole and ibandronate	15	HBSO, CHT	27 ANED
Horikawa et al. [39]	HBSO, MRM	No	N	Anastrozole, S-1	Concomitant	-	84 ANED
Komeda et al. [40]	MRM	4 × doxorubicin, cyclophosphamide/6 × paclitaxel	N	Letrozole	15	The patient refused HBSO, doxorubicin, and cyclophosphamide	28 DOD
Vicioso et al. [41]	Quadrantectomy, ALND.	6 × taxotere, adriamycin, and cyclophosphamide	N	Tamoxifen	72	HBSO, letrozole, and RT	163 AWD
Alligood-Percoco et al. [42]	MRM	4 × doxorubicin, 8 × CMF.	Y	Tamoxifen	116/240	HBSO, appendectomy, debulking, Taxotere, and Xeloda/Arimidex	240 AWD
Bezpalko et al. [43]	Biopsy	CHT	N	Hormonal therapy	Concomitant	-	1 DOD
Lokadasan et al. [44]	Biopsy	5-FU, epirubicin, cyclophosphamide	N	Hormonal therapy	Concomitant	-	NM
Lokadasan et al. [44]	MRM	3 × 5-FU, adriamycin, cyclophosphamide/3 × docetaxel	Y	Tamoxifen	48	Carboplatin and gemcitabine	NM
Toyoshima et al. [45]	Breast conserving surgery	6 × 5-FU, epirubicin, cyclophosphamide.	Y	Anastrozole	84	HBSO and exemestane	ANED
Waks et al. [46]	Breast conserving surgery, ALND	Methotrexate, cyclophosphamide, 5-FU	Y	Tamoxifen	180	Biopsy, CHT	NM
Lai et al. [47]	MRM	CHT	Y	Tamoxifen/anastrozole/exemestane	84	endometrial biopsy, CHT	AWD
Makris et al. [48]	Lumpectomy, ALND	6 × docetaxel, doxorubicin, cyclophosphamide	Y	No	24	HBSO, omentectomy, peritoneal biopsies, 6 × carboplatin, paclitaxel	18 ANED
Martinez et al. [49]	MRM, endometrial biopsy	CHT	N	Tamoxifen	Concomitant	-	
Martinez et al. [49]	MRM	CHT	Y	Tamoxifen	18	HBSO	NM
Akhtar et al. [50]	Biopsy	Patient refused CHT	N	N	Concomitant	-	Lost to follow-up
Bennett et al. [51]	NM	NM	NM	NM	NM	ΝΜ	49 DOD
Bennett et al. [51]	NM	NM	NM	NM	NM	ΝΜ	84 AWD
Bennett et al. [51]	NM	NM	NM	NM	NM	ΝΜ	9 DOD
Bennett et al. [51]	NM	NM	NM	NM	NM	ΝΜ	NM
Bennett et al. [51]	NM	NM	NM	NM	NM	ΝΜ	NM
Razia et al. [52]	Surgery	Doxifluridine, cyclophosphamide, and docetaxel	Y	Goserelin acetate, Tamoxifen, and toremifene citrate	108	HBSO, a partial colectomy, and an aromatase inhibitor	ANED
Seo et al. [53]	Breast conserving surgery, ALND	2 × neoadj. Cyclophosphamide, adriamycin 4 × adj. cyclophosphamide, adriamycin	Y	Goserelin and tamoxifen	24	HBSO	ANED
Aytekin et al. [54]	MRM	4 × adriamycin, cyclophosphamide/12 × paclitaxel	Y	Tamoxifen and luteinizing hormone-releasing hormone analog	10	HBSO and CHT	16 DOD
Briki et al. [55]	MRM	CHT	Y	Tamoxifen	24	HBSO	NM
Franko-Marquez et al. [56]	MRM	CHT	N	NM	360	Biopsy and CHT	NM
Kachi et al. [57]	MRM	9 × CHT	Y	Tamoxifen	60	Anterior resection, BO, and appendectomy	NM
Silva Fontinele et al. [58]	MRM	Neoadj. 4 × doxorubicin, and cyclophosphamide/12 × paclitaxel	Y	Tamoxifen	39	HBSO and anastrozole	66 ANED
Abdallah et al. [59]	HBSO	6 × cycolphosphamide, epirubicin 5-FU	N	Hormonal therapy	Concomitant	-	NM
Arif et al. [60]	NM	NM	N	Tamoxifen	84	HBSO	96 ANED
Gomez et al. [61]	MRM	NM	NM	Tamoxifen	60	Biopsy	NM
Yuan et al. [62]	MRM	CHT	Y	Hormonal therapy	132	Biopsy	ANED
Akizawa et al. [63]	HBSO, OM	Palbociclib and denosumab	N	Letrozole	Concomitant	-	ANED
Awazu et al. [64]	Surgery	CHT	NM	Aromatase inhibitors/tamoxifen	276	HBSO, partial OM, a biopsy of the peritoneum, fulvestrant, toremifene citrate, and tegafur	308 ANED
Lim et al. [65]	MRM	4 × cyclophosphamide, adriamycin, 5-FU/4 × taxotere	Y	Tamoxifen	30	HBSO, fulvestrant, and ribociclib	NM
Kong et al. [66]	MRM	4 × epirubicin and cyclophosphamide/4 × paclitaxel	patient refused RT	Letrozole	29	Radiotherapy, zoledronate/HBSO, 6 × paclitaxel, capecitabine, radiotherapy, and zoledronate/2 × gemcitabine, cisplatin	49 DOD
Li et al. [67]	MRM	6 × docetaxel, doxorubicin, cyclophosphamide	Y	Anastrozole	36	Hyperthermic perfusion chemotherapy (paclitaxel)	ANED
Benlghazi et al. [68]	MRM	3 × epirubicin, cyclophosphamide, and 5FU/3 × docetaxel	Y	Tamoxifen	60	HBSO and hormonal treatment	78 ANED
Faur et al. [69]	NM	NM	NM	NM	NM	NM	NM

Abbreviations: Adj.—adjuvant; adriamycin-cyclophosphamide (AC); ADR—adriamycin; ADM-TXL—doxorubicin (ADM), paclitaxel (TXL); ANED—alive no evidence of disease; AWD—alive with disease; BO—bilateral oophorectomy; cyclophosphamide-adriamycin-5-Fluorouracil (CAF); cyclophosphamide-methotrexate-5 Fluorouracil (CMF); CHT—chemotherapy (not specified); CTX—cyclophosphamide; docetaxel-doxorubicin-cyclophosphamide (TAC); DOD—died of disease; epirubicin-cyclophosphamide (EC); epirubicin-cyclophosphamide-5FU (FEC); FGT—female genital tract; 5-FU—5-fluorouracil; HBSO—hysterectomy and bilateral salpingo-oophorectomy; Met—metastasis; Mo—months; N—No; neoadj.—neoadjuvant; NM—not mentioned; OM—omentectomy; RT—radiotherapy; Y—Yes.

**Table 5:** Role of the study variables in patient overall survival. HR—hazard ratio, CI—confidence interval; N—number of valid cases.

Characteristic	HR and 95% CI	*p*-Value	N
Primary tumor ER status	0.41 (0.08–2.18)	0.283	22
Primary tumor PR status	0.27 (0.03–2.6)	0.223	22
Secondary tumor ER status	0.13 (0.02–0.8)	0.01	18
Secondary tumor PR status	2.04 (0.23–18.39)	0.518	16
HER-2 status	1.73 (0.11–27.89)	0.695	7
Stage pN	4.91 (0.2–118.15)	0.31	20
Stage pM	0.64 (0.07–5.76)	0.686	18
Other metastasis	0.82 (0.22–3.03)	0.76	31
RT	0.72 (0.17–3.02)	0.65	27

## Data Availability

This article is a review and not an original study. All the references are listed.

## References

[1] Cserni G., Floris G., Koufopoulos N., Kovács A., Nonni A., Regitnig P., Stahls A., Varga Z. (2017). Invasive lobular carcinoma with extracellular mucin production—A novel pattern of lobular carcinomas of the breast. Clinico-pathological description of eight cases. Virchows Arch..

[2] Lokuhetty D., White V.A., Watanave R., Cree I.A., WHO (2019). WHO Classification of Tumours. Breast Tumours.

[3] Koufopoulos N., Antoniadou F., Kokkali S., Pigadioti E., Khaldi L. (2019). Invasive lobular carcinoma with extracellular mucin production: Description of a case and review of the literature. Cureus.

[4] Koufopoulos N., Pateras I.S., Gouloumis A.R., Ieronimaki A.I., Zacharatou A., Spathis A., Leventakou D., Economopoulou P., Psyrri A., Arkadopoulos N. (2022). Diagnostically Challenging Subtypes of Invasive Lobular Carcinomas: How to Avoid Potential Diagnostic Pitfalls. Diagnostics.

[5] Reed A.E.M., Kutasovic J.R., Lakhani S.R., Simpson P.T. (2015). Invasive lobular carcinoma of the breast: Morphology, biomarkers and’omics. Breast Cancer Res..

[6] Koufopoulos N., Kokkali S., Antoniadou F., Dimas D.T., Missitzis I.L. (2019). Matrix-producing Breast Carcinoma: A Rare Subtype of Metaplastic Breast Carcinoma. Cureus.

[7] Mathew A., Rajagopal P.S., Villgran V., Sandhu G.S., Jankowitz R.C., Jacob M., Rosenzweig M., Oesterreich S., Brufsky A. (2017). Distinct pattern of metastases in patients with invasive lobular carcinoma of the breast. Geburtshilfe Frauenheilkd..

[8] Kioleoglou Z., Georgaki E., Koufopoulos N., Kostek O., Volakakis N., Dimitriadou A., Kokkali S. (2024). Gastrointestinal metastases from lobular breast carcinoma: A literature review. Cureus.

[9] Cserni G., Bori R., Ambrózay É., Serfőző O. (2024). Histological Patterns and Mammographic Presentation of Invasive Lobular Carcinoma Show No Obvious Associations. Cancers.

[10] Cserni G. (2024). Invasive lobular carcinoma of the breast: We diagnose it, but do we know what it is?. Pathologica.

[11] Christgen M., Cserni G., Floris G., Marchio C., Djerroudi L., Kreipe H., Derksen P.W.B., Vincent-Salomon A. (2021). Lobular Breast Cancer: Histomorphology and Different Concepts of a Special Spectrum of Tumors. Cancers.

[12] Cserni G. (2020). Histological type and typing of breast carcinomas and the WHO classification changes over time. Pathologica.

[13] Koufopoulos N.I., Boutas I., Pouliakis A., Samaras M.G., Kotanidis C., Kontogeorgi A., Dimas D.T., Ieronimaki A.I., Leventakou D., Spathis A. (2024). The “forgotten” subtypes of breast carcinoma: A systematic review of selected histological variants not included or not recognized as distinct entities in the current World Health Organization classification of breast tumors. Int. J. Mol. Sci..

[14] Koufopoulos N., Ieronimaki A.-I., Zacharatou A., Gouloumis A.R., Leventakou D., Boutas I., Dimas D.T., Kontogeorgi A., Sitara K., Khaldi L. (2023). A Case of Prostatic Signet-Ring Cell-like Carcinoma with Pagetoid Spread and Intraductal Carcinoma and Long-Term Survival: PD-L1 and Mismatch Repair System Proteins (MMR) Immunohistochemical Evaluation with Systematic Literature Review. J. Pers. Med..

[15] Christgen M., Steinemann D., Kühnle E., Länger F., Gluz O., Harbeck N., Kreipe H. (2016). Lobular breast cancer: Clinical, molecular and morphological characteristics. Pathol. Res. Pract..

[16] Aranda F.I., Laforga J.B., Martinez M.A. (1993). Metastasis from breast lobular carcinoma to an endometrial polyp Report of a case with immunohistochemical study. Acta Obstet. Gynecol. Scand..

[17] Sugiyama T., Toyoda N., Nose J., Kihira N., Ando Y., Ishihara A. (1995). Breast cancer metastatic to uterine leiomyoma: A case report. J. Obstet. Gynaecol..

[18] Menzin A.W., De Risi D., Smilari T.F., Kalish P.E., Vinciguerra V. (1998). Lobular breast carcinoma metastatic to the vulva: A case report and literature review. Gynecol. Oncol..

[19] Arnould L., Franco N., Soubeyrand M.S., Mege F., Belichard C., Lizard-Nacol S., Collin F. (2002). Breast carcinoma metastasis within granulosa cell tumor of the ovary: Morphologic, immunohistologic, and molecular analyses of the two different tumor cell populations. Hum. Pathol..

[20] Alvarez C., Ortiz-Rey J., Estévez F., De La Fuente A. (2003). Metastatic lobular breast carcinoma to an endometrial polyp diagnosed by hysteroscopic biopsy. Obstet. Gynecol..

[21] Ogino A., Nomizu T., Gonnda K., Okouchi C., Sakuma T., Yamada M., Katagata N., Watanabe F., Yamaguchi Y., Yoshida T. (2003). A case of breast cancer metastasizing to cervix after resection of pancreatic metastasis. Breast Cancer.

[22] Rau A.R., Saldanha P., Raghuveer C. (2003). Metastatic lobular mammary carcinoma diagnosed in cervicovaginal smears: A case report. Diagn. Cytopathol..

[23] Blecharz P., Szpor J., Karolewski K., Ryś J. (2004). Breast cancer metastases to uterine leiomyomas-a clinical and patomorphological analysis of two cases. Nowotw. J. Oncol..

[24] Famoriyo A., Sawant S., Banfield P.J. (2004). Abnormal uterine bleeding as a presentation of metastatic breast disease in a patient with advanced breast cancer on tamoxifen therapy. Arch. Gynecol. Obstet..

[25] Sheen-Chen S.M., Eng H.L., Huang C.C. (2004). Breast cancer metastatic to the vulva. Gynecol. Oncol..

[26] Al-Brahim N., Elavathil L.J. (2005). Metastatic breast lobular carcinoma to tamoxifen-associated endometrial polyp: Case report and literature review. Ann. Diagn. Pathol..

[27] Lee T.F., Wang Y.L., Wei T.S., Chen C.P. (2005). Incidental detection of metastatic lobular breast carcinoma in the female internal genital organs 2 years following modified radical mastectomy. Taiwan. J. Obstet. Gynecol..

[28] Scopa C.D., Aletra C., Lifschitz-Mercer B., Czernobilsky B. (2005). Metastases of breast carcinoma to the uterus. Report of two cases, one harboring a primary endometrioid carcinoma, with review of the literature. Gynecol. Oncol..

[29] Chen P., Hu W.M., Wang P.H., Suen J.H. (2006). Recurrent breast cancer presents as a single solid ovarian mass and ascites. Taiwan. J. Obstet. Gynecol..

[30] Erkanli S., Kayaselcuk F., Kuscu E., Bolat F., Sakalli H., Haberal A. (2006). Lobular carcinoma of the breast metastatic to the uterus in a patient under adjuvant anastrozole therapy. Breast.

[31] Perišić D., Jančić S., Kalinović D., Čekerevac M. (2007). Metastasis of lobular breast carcinoma to the cervix. J. Obstet. Gynaecol. Res..

[32] Manci N., Marchetti C., Esposito F., Graziano M., Tomao F., Pastore M., Bellati F., Panici P.B. (2008). Late breast cancer recurrence to the uterine cervix with a review of the literature. Int. J. Gynecol. Pathol..

[33] Manipadam M., Walter N., Selvamani B. (2008). Lobular carcinoma metastasis to endometrial polyp unrelated to tamoxifen: Report of a case and review of the literature. Apmis.

[34] Bogliolo S., Morotti M., Valenzano Menada M., Fulcheri E., Musizzano Y., Casabona F. (2010). Breast cancer with synchronous massive metastasis in the uterine cervix: A case report and review of the literature. Arch. Gynecol. Obstet..

[35] Ustaalioglu B.B., Bilici A., Seker M., Salman T., Gumus M., Barisik N.O., Salepci T., Yaylaci M. (2009). Metastasis of lobular breast carcinoma to the uterus in a patient under anastrozole therapy. Oncologie.

[36] Engelstaedter V., Mylonas I. (2011). Lower genital tract metastases at time of first diagnosis of mammary invasive lobular carcinoma. Arch. Gynecol. Obstet..

[37] Hooker A., Radder C., van De Wiel B., Geenen M. (2011). Metastasis from breast cancer to an endometrial polyp; treatment options and follow-up. Report of a case and review of the literature. Eur. J. Gynaecol. Oncol..

[38] Işçi H., Güdücü N., Basgul A., Aydınlı K., Calay Z., Dünder I. (2011). Lobular carcinoma of the breast metastasızıng to leiomyoma in a patient under letrozole treatment. Eur. J. Gynaecol. Oncol..

[39] Horikawa M., Mori Y., Nagai S., Tanaka S., Saito S., Okamoto T. (2012). Metastatic breast cancer to the uterine cervix mimicking a giant cervical leiomyoma. Nagoya J. Med. Sci..

[40] Komeda S., Furukawa N., Kasai T., Washida A., Kobayashi H. (2013). Uterine metastasis of lobular breast cancer during adjuvant letrozole therapy. J. Obstet. Gynaecol..

[41] Vicioso L., Ortega M.V., Cívico V., López-Beltrán A. (2013). Synchronous metastasis from lobular carcinoma and primary carcinoma of the endometrium in a patient after tamoxifen therapy. Int. J. Gynecol. Pathol..

[42] Alligood-Percoco N.R., Kessler M.S., Willis G. (2015). Breast cancer metastasis to the vulva 20 years remote from initial diagnosis: A case report and literature review. Gynecol. Oncol. Rep..

[43] Bezpalko K., Mohamed M.A., Mercer L., McCann M., Elghawy K., Wilson K. (2015). Concomitant endometrial and gallbladder metastasis in advanced multiple metastatic invasive lobular carcinoma of the breast: A rare case report. Int. J. Surg. Case Rep..

[44] Lokadasan R., Ratheesan K., Sukumaran R., Nair S.P. (2015). Metastatic lobular carcinoma of breast mimics primary cervix carcinoma: Two case reports and a review of the literature. Ecancermedicalscience..

[45] Toyoshima M., Iwahashi H., Shima T., Hayasaka A., Kudo T., Makino H., Igeta S., Matsuura R., Ishigaki N., Akagi K. (2015). Solitary uterine metastasis of invasive lobular carcinoma after adjuvant endocrine therapy: A case report. J. Med. Case Rep..

[46] Waks A.G., Lennon J., Yadav B.S., Hwang H., dSchapirael Carmen M., Johnson N.B., Reynolds K., Schapira L., Gilman P.B., Overmoyer B. (2015). Metastasis to the Cervix Uteri 15 Years After Treatment of Lobular Carcinoma of the Breast. Semin. Oncol..

[47] Lai M.J., Lai C.L., Huang I.H., Yu J.C., Lee H.S., Dai M.S. (2016). Synchronous endometrial and gastric metastases of invasive lobular breast carcinomas. Taiwan. J. Obstet. Gynecol..

[48] Makris G.M., Marinelis A., Battista M.J., Chrelias C., Papantoniou N. (2017). An ovarian mass after breast cancer: Metachronous carcinoma or metastasis? A case report. Int. J. Surg. Case Rep..

[49] Martinez M.R., Marazuela M.A., Vallejo M.R., Bernabeu R.Á., Medina T.P. (2016). Metastasis of lobular breast cancer to endometrial polyps with and without the presence of vaginal bleeding. Int. J. Gynecol. Obstet..

[50] Akhtar A., Ratra A., Puckett Y., Sheikh A.B., Ronaghan C.A. (2017). Synchronous uterine metastases from breast cancer: Case study and literature review. Cureus.

[51] Bennett J.A., Young R.H., Chuang A.Y., Lerwill M.F. (2018). Ovarian metastases of breast cancers with signet ring cells: A report of 17 cases including 14 Krukenberg tumors. Int. J. Gynecol. Pathol..

[52] Razia S., Nakayama K., Tsukao M., Nakamura K., Ishikawa M., Ishibashi T., Ishikawa N., Sanuki K., Yamashita H., Ono R. (2017). Metastasis of breast cancer to an endometrial polyp, the cervix and a leiomyoma: A case report and review of the literature. Oncol. Lett..

[53] Seo S.O., Shin J.Y., Ji Y.I. (2017). Metastatic uterine cancer looking as cervical fibroid in recurrent breast cancer woman: A case report. Obstet. Gynecol. Sci..

[54] Aytekin A., Bilgetekin I., Ciltas A., Ogut B., Coskun U., Benekli M. (2018). Lobular breast cancer metastasis to uterus during adjuvant tamoxifen treatment: A case report and review of the literature. J. Cancer Res. Ther..

[55] Briki R., Cherif O., Bannour B., Hidar S., Boughizane S., Khairi H. (2018). Uncommon metastases of invasive lobular breast cancer to the endometrium: A report of two cases and review of the literature. Pan Afr. Med. J..

[56] Franco-Márquez R., Torres-Gaytán A.G., Narro-Martinez M.A., Carrasco-Chapa A., Núñez B.G., Boland-Rodriguez E. (2019). Metastasis of Breast Lobular Carcinoma to Endometrium Presenting as Recurrent Abnormal Uterine Bleeding: A Case Report and Review of Literature. Case Rep. Pathol..

[57] Kachi A., Nicolas G., Semaan D.B., Hashem M., Abou Sleiman C. (2019). Unusual pattern of invasive lobular carcinoma metastasis: A case report. Am. J. Case Rep..

[58] Fontinele D.R.S., Vieira S.C., da Silva Júnior R.G., Rodrigues T.S. (2019). Lobular carcinoma of the breast with metastasis to the uterine cervix. J. Cancer Res. Ther..

[59] Abdallah H., Elwy A., Alsayed A., Rabea A., Magdy N. (2020). Metastatic breast lobular carcinoma to unusual sites: A report of three cases and review of literature. J. Med. Cases.

[60] Arif S.H., Mohammed A.A., Mohammed F.R. (2020). Metastatic invasive lobular carcinoma of the breast to the endometrium presenting with abnormal uterine bleeding; Case report. Ann. Med. Surg..

[61] Gomez M., Whitting K., Naous R. (2020). Lobular breast carcinoma metastatic to the endometrium in a patient under tamoxifen therapy: A case report. SAGE Open Med. Case Rep..

[62] Yuan L., Oshilaja O., Sierk A., Zhang G., Booth C.N., Brainard J., Dyhdalo K.S. (2021). Metastatic breast cancer diagnosed on cervical cytology. Cytopathology.

[63] Akizawa Y., Kanno T., Horibe Y., Shimizu Y., Noguchi E., Yamamoto T., Okamoto T., Nagashima Y., Tabata T. (2021). Ovarian metastasis from breast cancer mimicking a primary ovarian neoplasm: A case report. Mol. Clin. Oncol..

[64] Awazu Y., Fukuda T., Imai K., Yamauch M., Kasai M., Ichimura T., Yasui T., Sumi T. (2021). Uterine metastasis of lobular breast carcinoma under tamoxifen therapy: A case report. Mol. Clin. Oncol..

[65] Lim L., Wang T.Y., Lam H.B., Chang C.L. (2021). Massive metastasis of breast cancer to female genital organs. Taiwan. J. Obstet. Gynecol..

[66] Kong D., Dong X., Qin P., Sun D., Zhang Z., Zhang Y., Hao F., Wang M. (2022). Asymptomatic uterine metastasis of breast cancer: Case report and literature review. Medicine.

[67] Li T., Jiang X., Zhang Z., Chen X., Wang J., Zhao X., Zhang J. (2022). Case Report: 68Ga-FAPI PET/CT, a more advantageous detection mean of gastric, peritoneal, and ovarian metastases from breast cancer. Front. Oncol..

[68] Benlghazi A., Messaoudi H., Benali S., Tazi I., Elhassani M.M., Kouach J. (2024). Lobular carcinoma metastasis to endometrial polyps: Insights from a case report and literature analysis. Int. J. Surg. Case Rep..

[69] Faur A.C., Gurban C.V., Dăescu E., Tîrziu R.V., Lazăr D.C., Ghenciu L.A. (2024). Mucin-Producing Lobular Breast Carcinoma Metastasis to an Ovarian Fibroma: Histopathological and Immunohistochemical Analysis of a Rare Case and Literature Review. Diagnostics.

[70] Mazur M.T., Hsueh S., Gersell D.J. (1984). Metastases to the female genital tract: Analysis of 325 cases. Cancer.

[71] Harris M., Howell A., Chrissohou M., Swindell R., Hudson M., Sellwood R. (1984). A comparison of the metastatic pattern of infiltrating lobular carcinoma and infiltrating duct carcinoma of the breast. Br. J. Cancer.

[72] Lamovec J., Bračkko M. (1991). Metastatic pattern of infiltrating lobular carcinoma of the breast: An autopsy study. J. Surg. Oncol..

[73] Winston C.B., Hadar O., Teitcher J.B., Caravelli J.F., Sklarin N.T., Panicek D.M., Liberman L. (2000). Metastatic lobular carcinoma of the breast: Patterns of spread in the chest, abdomen, and pelvis on CT. AJR Am. J. Roentgenol..

[74] Ayhan A., Guvenal T., Salman M., Ozyuncu O., Sakinci M., Basaran M. (2005). The role of cytoreductive surgery in nongenital cancers metastatic to the ovaries. Gynecol. Oncol..

[75] Tian W., Zhou Y., Wu M., Yao Y., Deng Y. (2019). Ovarian metastasis from breast cancer: A comprehensive review. Clin. Transl. Oncol..

[76] Bigorie V., Morice P., Duvillard P., Antoine M., Cortez A., Flejou J.F., Uzan S., Darai E., Barranger E. (2010). Ovarian metastases from breast cancer: Report of 29 cases. Cancer Interdiscip. Int. J. Am. Cancer Soc..

[77] Bastings L., Beerendonk C., Westphal J., Massuger L.F., Kaal S.E., van Leeuwen F.E., Braat D.D., Peek R. (2013). Autotransplantation of cryopreserved ovarian tissue in cancer survivors and the risk of reintroducing malignancy: A systematic review. Hum. Reprod. Update.

[78] Peters I.T., van Zwet E.W., Smit V.T., Liefers G.J., Kuppen P.J., Hilders C.G., Trimbos J.B. (2017). Prevalence and risk factors of ovarian metastases in breast cancer patients <41 years of age in the Netherlands: A nationwide retrospective cohort study. PLoS ONE..

[79] He H., Gonzalez A., Robinson E., Yang W.T. (2014). Distant metastatic disease manifestations in infiltrating lobular carcinoma of the breast. AJR Am. J. Roentgenol..

[80] Guerriero S., Alcazar J., Pascual M., Ajossa S., Olartecoechea B., Hereter L. (2012). Preoperative diagnosis of metastatic ovarian cancer is related to origin of primary tumor. Ultrasound Obstet. Gynecol..

[81] Boutas I., Kontogeorgi A., Koufopoulos N., Dimas D.T., Sitara K., Kalantaridou S.N., Dimitrakakis K. (2023). Breast cancer and fertility preservation in young female patients: A systematic review of the literature. Clin. Pract..

[82] Webb M.J., Decker D.G., Mussey E. (1975). Cancer metastatic to the ovary: Factors influencing survival. Obstet. Gynecol..

[83] Fujiwara K., Ohishi Y., Koike H., Sawada S., Moriya T., Kohno I. (1995). Clinical implications of metastases to the ovary. Gynecol. Oncol..

[84] Moore E., Roylance R., Rosenthal A. (2012). Breast cancer metastasising to the pelvis and abdomen: What the gynaecologist needs to know. BJOG.

[85] Nandy S.B., Gangwani L., Nahleh Z., Subramani R., Arumugam A., de la Rosa J.M., Lakshmanaswamy R. (2015). Recurrence and metastasis of breast cancer is influenced by ovarian hormone’s effect on breast cancer stem cells. Future Oncol..

[86] de la Monte S.M., Hutchins G.M., Moore G.W. (1988). Influence of age on the metastatic behavior of breast carcinoma. Hum. Pathol..

[87] Pimentel C., Becquet M., Lavoué V., Hénno S., Lévêque J., Ouldamer L. (2016). Ovarian metastases from breast cancer: A series of 28 cases. Anticancer. Res..

[88] Abd El hafez A., Monir A. (2013). Diagnostic spectrum of ovarian masses in women with breast cancer; magnetic resonance imaging: Histopathology correlation. Ann. Diagn. Pathol..

[89] Curtin J.P., Barakat R.R., Hoskins W.J. (1994). Ovarian disease in women with breast cancer. Obstet. Gynecol..

[90] Abu-Rustum N.R., Aghajanian C.A., Venkatraman E.S., Feroz F., Barakat R.R. (1997). Metastatic breast carcinoma to the abdomen and pelvis. Gynecol. Oncol..

[91] Rabban J.T., Barnes M., Chen L.M., Powell C.B., Crawford B., Zaloudek C.J. (2009). Ovarian pathology in risk-reducing salpingo-oophorectomies from women with BRCA mutations, emphasizing the differential diagnosis of occult primary and metastatic carcinoma. Am. J. Surg. Pathol..

[92] Perrotin F., Marret H., Bouquin R., Fischer-Perrotin N., Lansac J., Body G. (2001). Incidence, diagnosis and prognosis of ovarian metastasis in breast cancer. Gynecol. Obstet. Fertil..

[93] Antila R., Jalkanen J., Heikinheimo O. (2006). Comparison of secondary and primary ovarian malignancies reveals differences in their pre-and perioperative characteristics. Gynecol. Oncol..

[94] Bruls J., Simons M., Overbeek L.I., Bulten J., Massuger L.F., Nagtegaal I.D. (2015). A national population-based study provides insight in the origin of malignancies metastatic to the ovary. Virchows Arch..

[95] Kubeček O., Laco J., Špaček J., Petera J., Kopecký J., Kubečková A., Filip S. (2017). The pathogenesis, diagnosis, and management of metastatic tumors to the ovary: A comprehensive review. Clin. Exp. Metastasis.

[96] Yadav B.S., Sharma S., Robin T.P., Sams S., Elias A.D., Kaklamani V., Kelly Marcom P., Schaefer S., Morris G.J. (2015). Synchronous primary carcinoma of breast and ovary versus ovarian metastases. Semin. Oncol..

[97] Tamás J., Vereczkey I., Tóth E. (2015). Metastatic tumors in the ovary, difficulties of histologic diagnosis. Magy. Onkol..

[98] Zhang R., Liu J., Jiang L., Lang Z. (2025). Application of TRPS1 in ER-negative or low expression distant metastatic breast carcinoma. Pathol. Oncol. Res..

[99] Zafrakas M., Petschke B., Donner A., Fritzsche F., Kristiansen G., Knüchel R., Dahl E. (2006). Expression analysis of mammaglobin A (SCGB2A2) and lipophilin B (SCGB1D2) in more than 300 human tumors and matching normal tissues reveals their co-expression in gynecologic malignancies. BMC Cancer.

[100] Pauer H.U., Viereck V., Burfeind P., Emons G., Krauss T. (2003). Uterine cervical metastasis of breast cancer: A rare complication that may be overlooked. Oncologie.

[101] Pérez-Montiel D., Serrano-Olvera A., Salazar L.C., Cetina-Pérez L., Candelaria M., Coronel J., Montalvo L.A., de León D.C. (2012). Adenocarcinoma metastatic to the uterine cervix: A case series. J. Obstet. Gynaecol. Res..

[102] Abell M.R., Gosling J.R. (1962). Gland cell carcinoma (adenocarcinoma) of the uterine cervix. Am. J. Obstet. Gynecol..

[103] Scott K., Bryson G., Jamison J., Coutts M., McCluggage W.G. (2018). Cervical squamous carcinomas with prominent acantholysis and areas resembling breast lobular carcinoma: An aggressive form of dedifferentation. Int. J. Gynecol. Pathol..

[104] Mansor S., McCluggage W.G. (2010). Cervical adenocarcinoma resembling breast lobular carcinoma: A hitherto undescribed variant of primary cervical adenocarcinoma. Int. J. Gynecol. Pathol..

[105] Hermi A., Chakroun M., Saadi A., Saidani B., Kacem L.B., Chebil M. (2022). Upper urinary tract urothelial carcinoma diagnosis by biopsy of a vaginal metastasis. Urol. Case Rep..

[106] Yan Y., Guo T., Zhang M., Cui G. (2023). Vaginal metastasis from breast cancer: A case report. Open Life Sci..

[107] Gandhi A.K., Roy S., Mridha A.R., Sharma D.N. (2015). Vulvar metastasis from carcinoma breast unveiling distant metastasis: Exploring an unusual metastatic pattern. J. Egypt. Natl. Cancer Inst..

